# Older injured adults reported sleep disturbances: a cross-sectional study

**DOI:** 10.1186/s41687-025-00974-2

**Published:** 2025-11-25

**Authors:** Chris Azar, Cristina Barboi, Anthony Perkins, Damaris Ortiz, Ben L. Zarzaur, Abdelfattah Alhader, Malaz A. Boustani

**Affiliations:** 1 Department of Medicine, Division of General Internal Medicine and Geriatrics, Indiana University School of Medicine, Indianapolis, IN USA; 2 Department of Anesthesiology, Indiana University School of Medicine, Indianapolis, IN USA; 3 Department of Biostatistics and Health Data Science, Indiana University School of Medicine, Indianapolis, IN USA; 4 Department of Surgery, Indiana University School of Medicine, Indianapolis, IN USA; 5 Department of Surgery, University of Wisconsin School of Medicine and Public Health, Madison, WI USA; 6 Department of Physiology and Biochemistry, Faculty of Medicine, Jordan University of Science and Technology, P.O. Box 3030, Irbid, 22110 Jordan; 7 Center for Health Innovation and Implementation Science, Indiana University School of Medicine, Indianapolis, IN USA

**Keywords:** Sleep disturbances, Post traumatic stress disorder, Traumatic injury

## Abstract

**Background:**

Sleep disturbances are common among older adults recovering from traumatic injury and are associated with delayed psychological and functional recovery. Despite their prevalence, few studies have examined sleep disturbances early after injury in this population. This study evaluated the prevalence of self-reported sleep disturbances among older trauma survivors using PROMIS-based assessments within the Trauma Medical Home (TMH) care model to identify modifiable factors that may inform early, patient-centered interventions.

**Methodology:**

In this cross-sectional study, we conducted a secondary analysis of data from the Collaborative Care for Injured Older Adults: The Trauma Medical Home Randomized Clinical Trial (TMH) study. 144 patients 50 years of age and older admitted for traumatic injury into four trauma centers reported their sleep quality using Patient-Reported Outcomes Measurement Information System Sleep Disturbance Short Form 4a (PROMIS-SF). Symptoms of depression and anxiety were assessed using the Hospital Anxiety and Depression Scale (HADS), of post-traumatic stress using Post Traumatic Stress Disorder (PTSD) Checklist-Civilian Version (PCL-C), and pain intensity using Pain, Enjoyment of Life, and General Activity Scale (PEG) scale. Comorbidities were quantifiedusing the Charlson Comorbidity Index (CCI), and trauma severity using the Injury Severity Score (ISS). Logistic regression was used to examine associations between sleep disturbances and psychological and clinical factors.

**Results:**

Approximately 22% of patients reported sleep disturbances. Patients with PTSD or pain symptoms had significantly higher odds of reporting sleep disturbances (OR for PTSD = 13.21, 95% CI = [2.90, 61.17], *p* = 0.001; OR for pain = 1.33, 95% CI = [1.11, 1.60], *p* = 0.002).

**Conclusion:**

This study extends prior research by focusing on older adult trauma survivors and assessing sleep disturbances during the immediate post-injury period using PROMIS-based patient-reported outcomes within the Trauma Medical Home (TMH) care model. This approach provides new insight into early, multidimensional symptom patterns and identifies modifiable factors that can inform timely, patient-centered interventions to improve recovery.

## Introduction

More than 50% of older adult injury survivors are admitted to the Intensive Care Unit (ICU) having sleep disturbances [1–5]. Deterioration in sleep quality is three times more prevalent among injury survivors compared to the general population, and 60% of these patients have long-term symptoms [6]. Sleep disturbances persist for up to twelve months post-injury [3, 4], disrupting the cognitive, functional, and psychological recovery of survivors with a significant financial burden [1–7].

Sleep plays a vital role in maintaining the physiologic homeostasis of the metabolic, immune, hormonal, and cardiovascular systems [8, 9]. Numerous adverse health outcomes and disorders have been linked to psychological changes related to sleep disorders [10]. Sleep deprivation and sleep disorders have been associated with an increased risk of hypertension, diabetes, obesity, depression, heart attack, and stroke [8]. Psychosocial and cognitive impairmentssuch as emotional distress, deficits in cognitive performance and memory function, depression, and suicidal ideation are also linked to disrupted sleep [10–12]. Among individuals with PTSD, sleep disturbance is one of the most frequently reported symptoms and is associated with greater functional disability and prolonged recovery. Preexisting or comorbid sleep problems may exacerbate PTSD symptoms and complicate rehabilitation [13, 14].

A substantial body of evidence supports the association between self-reported sleep disturbances and PTSD [15, 16] with estimates of up to 80–90% of patients with PTSD experiencing insomnia symptoms [17]. The prevalence of PTSD among patients hospitalized for acute traumatic injuries ranges between 13% and 51%^17^. Prior research has demonstrated bidirectional relationships between sleep disturbances and psychological symptoms such as depression, anxiety, and PTSD, as well as pain following trauma [18–20]. When hyperarousal, nightmares, or intrusive thoughts occur, they can fragment the sleep cycle and contribute to chronic insomnia. In turn, fear of sleep or trauma-related avoidance behaviors may perpetuate these disturbances [21]. Sleep disturbances are considered a modifiable risk factor for the onset and relapse of psychiatric disorders. Addressing sleep problems early after trauma may prevent psychological deteriorations and improve recovery outcomes [20, 22].

Patient-reported outcomes provide valuable insight into symptom burden, helping clinicians prioritize patient concerns, identify individuals who may benefit from diagnostic testing, and tailor interventions to specific needs. Older adults recovering from trauma are particularly vulnerable to long-term biopsychosocial dysfunction, with recovery trajectories that vary widely among individuals [23]. In prior work, our team developed and evaluated the Trauma Medical Home (TMH) - a collaborative care model designed to address the complex needs of older (≥ 50 years) trauma survivors and enhance their biopsychosocial functioning [24]. This study aimed to evaluate the prevalence of self-reported sleep disturbances and appraise associated factors using a Patient-Reported Outcomes Measurement Information System (PROMIS) assessment.

We performed a secondary analysis of the TMH data to evaluate the prevalence of self-reported sleep disturbances and their associations with pain, depression, anxiety, and post-traumatic stress symptoms in older trauma survivors during the immediate post-injury period. We hypothesized that in older injured adults, pain and psychological symptoms would be associated with self-reported sleep disturbances. Because patients were assessed during hospitalization in the post-acute phase, we considered sleep disturbances to reflect the early outcomes of trauma exposure and associated psychological symptoms. The novelty of our approach lies in the combination of three elements: the focus on older adult trauma survivors, the early timing of post-injury assessment, and the integration of PROMIS measures within the Trauma Medical Home (TMH) framework. By combining multidimensional *PROMIS* symptom assessment with a patient-centered care model, this study provides novel insight into *early post-injury* sleep disturbances *among older adults*. These findings will advance understanding of the biopsychosocial determinants of recovery.

Understanding patient-reported factors associated with sleep disturbance in this population may help identify targets for tailored interventions to improve recovery and quality of life.

## Methods

### Aim, study design, and settings

This is a cross-sectional study of patient-reported sleep quality in older injured adults, resulting from a secondary analysis of participant data collected during the *Collaborative Care of Older Adults in the Trauma Medical Home (TMH*) study. The TMH study is a single-blinded, randomized clinical trial that evaluated the efficacy of collaborative care of older adults in the Trauma Medical Home (TMH) at 4 level I trauma centers between October 11, 2017, and October 13, 2021. The TMH study intervention was delivered by an interdisciplinary team and addressed biopsychosocial domains impacted by injury, including cognition, anxiety, depression, sleep, and physical function. TMH participants with high anxiety or depression scores at baseline randomized to the intervention group, experienced improved Mental Component Summary scores in the follow-up Short Form-36 assessments of quality of life. The protocol and main study results of the clinical trial have been published [24, 25]. The Indiana University Institutional Review Board approved the study (IRB #1612690852).

### Participants

This study includes 216 participants, aged 50 years and older, who were hospitalized with a physical injury and an Injury Severity Score (ISS) of 9 or higher, and had access to a telephone following hospital discharge. Only participants in the TMH intervention group were included in this study; the usual care group from TMH did not complete sleep quality assessments. The baseline assessment was performed at enrollment and during hospitalization. The TMH intervention was delivered after hospital discharge by a nurse care coordinator guided by an interdisciplinary team (trauma surgeon, pulmonary critical care, geriatrician physicians, nurses, and psychologist) in partnership with primary care and addressed biopsychosocial domains impacted by injury, including cognition, anxiety, depression, sleep, and physical function. Exclusion criteria included significant head injury (indicated by intracranial bleeding on imaging or a Glasgow Coma Scale score below 13), spinal cord injury with a persistent neurological impairment upon hospital release, stroke either on admission or during hospitalization, baseline cognitive impairment (noted by any reference to dementia or Alzheimer’s disease in medical records), sensory impairments that hinder active engagement with study evaluations or communications, burns affecting over 10% of total body surface area, recent drug or alcohol use disorder (identified by medical records and/or the Alcohol Use Disorders Screening Test C or Drug Abuse Screening Test within the last 6 months), incarceration, malignancy with a life expectancy of less than 1 year, or residence located more than 50 miles away from the admitting trauma facility.

### Patients demographic characteristics, clinical factors, and reported outcome measures

The participants’ demographic information was collected at enrollment and included race, ethnicity, sex, and years of education completed.

The clinical factors were collected from the Electronic Medical Records (EMR) and included patients’ medical history stratified with the Charlson Comorbidity Index (CCI) and the Injury Severity Score (ISS). CCI score of zero indicates that no comorbidities are present; mild comorbidities are indicated by a score of 1–2; moderate comorbidities by a score of 3–4; and severe comorbidities by a score higher than 5 [26]. CCI predicts patients’ ten-year mortality [27]; the higher the score, the more likely the predicted outcome will result in mortality or higher resource utilization. The type of injury was noted, and the injury severity was evaluated using the Injury Severity Score (ISS), with a threshold of 15 indicating severe trauma.

*The Patient-Reported Outcomes Measurements* (PROM) were collected at the time of baseline evaluation by a nurse coordinator using validated tools that measure sleep disturbances, symptoms of depression, anxiety, traumatic stress, and pain. *The Patient-Reported Outcomes Measurement Information System Sleep Disturbance Short Form 4a (PROMIS-SF)* with a maximum raw score of 20 and a minimum score of 4, evaluated sleep quality after hospitalization for trauma [28]. This tool measures the depth, quality, and satisfaction of the patient’s sleep during the preceding seven days and has been validated for use in hospitalized and acutely ill populations [28]. The 7-day recall aligns with the PROMIS instrument’s intended design and provides a reliable representation of recent sleep experiences, offering clinically meaningful data on sleep quality during early recovery [28]. PROMIS measures capture patients’ perceived sleep experience rather than single night variability making them suitable for assessing sleep patterns in the post-injury phase [29]. For this analysis, based on prior literature, a raw score of ≥ 14 was used to define clinically significant sleep disturbance [30, 31].

*The depression and anxiety subdomains of the Hospital Anxiety and Depression Scale (HADS*) screened for anxiety and depression at the time of hospitalization [32]. Each of the seven items in the anxiety and depression subdomains has a value ranging from 0 to 3, for a total score ranging from 0 to 21^1^; a score of 8 or higher in either subdomain indicated a positive screen for depression or anxiety symptoms.

*The PTSD Checklist-Civilian Version (PCL-C)*, with a range of 17–85, screened for PTSD at the time of hospitalization; a score below 28–29 indicates little to no severity [18]. For this study, a score greater than or equal to 30 indicated a positive screen for traumatic stress symptoms [33].

*The Pain*,* Enjoyment of Life*,* and General Activity Scale (PEG)* rated pain intensity (range of 0–10) and its effects on everyday activities and quality of life at admission. A higher score corresponds to a larger burden of pain [34]. The scores were used as a continuous variable.

### Analysis

Data distributions were examined for all variables prior to analysis. The Charlson Comorbidity Index (CCI) demonstrated a skewed distribution and was therefore analyzed using a non-parametric test; CCI was excluded from the multivariable logistic regression model. All variables included in the regression were categorical and normally distributed, satisfying model assumptions. Participants’ demographic and clinical characteristics were summarized using frequencies and percentages for categorical variables, means, and standard deviations (SD) for continuous variables. Fisher’s exact tests were used to evaluate differences in categorical characteristics by the presence or absence of a sleep disturbance. Two-sample T-tests were used for comparisons of normally distributed continuous variables, and Wilcoxon rank-sum tests were applied for skewed data. A logistic regression model was constructed with sleep disturbance (defined as a PROMIS-SF raw score ≥ 14) as the dependent variable. Demographic and clinical variables that demonstrated significant associations with sleep disturbance in bivariate analyses were included as independent variables in the multivariable model. Statistical significance was set at an alpha level of 0.05. All analyses were performed using SAS v9.4.

## Results

### Patient characteristics

Table 1 shows the overall characteristics of this study’s patients. In the TMH trial, 216 participants received the intervention. Of these, 144 (66.7%) completed the PROMIS Sleep Disturbance Short Form 4a. 52.8% of the study population were female. All participants were over 50, and 79.1% were between 50 and 79. Of the injuries encountered, 56% were caused by falls, followed by motor vehicle crashes (29%). The mean injury severity score was 11.8(SD = 3.8). The mean CCI was 1 (SD = 1.6). Approximately 10% of patients had symptoms of depression and PTSD, 9% had symptoms of anxiety, and 21.5% had sleep disturbance.

A comparison of demographic and clinical characteristics among patients with and without sleep disturbance is presented in Table 2. Patients with a sleep disturbance were younger (50–64 years old) and more likely to have symptoms of depression (*p* < 0.013), anxiety (*p* < 0.001), and PTSD (*p* < 0.001) compared to patients without a sleep disturbance. Patients with a sleep disturbance reported significantly higher pain than patients without a sleep disturbance (*p* < 0.001).

**Table 1:** Distribution of overall patient characteristics (*n* = 144)

	% (*n*) or Mean (SD)
**Age, % (n)**	
50–64	34.0 (49)
65–79	45.1 (65)
80+	20.8 (30)
Female, % (n)	52.8 (76)
Hispanic, % (n)	0.7 (1)
**Race, % (n)**	
Black	9.1 (13)
Other	1.4 (2)
White	89.5 (128)
**Mechanism of Injury**,** % (n)**	
Fall	55.6 (80)
Motor Vehicle Accident	29.2 (42)
Other	15.3 (22)
Mean ISS(SD)	11.8 (3.8)
Mean CCI (SD)	1.0 (1.6)
Mean PEG (SD)	4.4 (2.7)
**HADS**	
Depression, % (n)	9.8 (14)
Anxiety, % (n)	9.1 (13)
PTSD, % (n)	10.4 (15)
Sleep Disturbance, % (n)	21.5 (31)

CCI, Charlson comorbidity Index; PEG, Pain, enjoyment of Life, and general activity Scale; ISS, injury severity Score; HADS, hospital anxiety and depression Scale; PTSD, Post-Traumatic stress syndrome

**Table 2:** Comparison of demographic and clinical characteristics by presence of sleep disturbance

	No sleep disturbance (*n* = 113)	Sleep disturbance (*n* = 31)	*P*-value
**Age**,** % (n)**			0.049
50–64	29.2 (33)	51.6 (16)	
65–79	46.9 (53)	38.7 (12)	
80+	23.9 (27)	9.7 (3)	
Female, % (n)	54.0 (61)	48.4 (15)	0.686
Hispanic, % (n)	0.0 (0)	3.2 (1)	0.220
**Race**,** % (n)**			0.683
Black	8.0 (9)	12.9 (4)	
Other	1.8 (2)	0.0 (0)	
White	90.2 (101)	87.1 (27)	
**Mechanism of Injury**,** % (n)**			0.245
Fall	58.4 (66)	45.2 (14)	
Motor Vehicle Accident	25.7 (29)	41.9 (13)	
Other	15.9 (18)	12.9 (4)	
Mean ISS (SD)	11.6 (3.9)	12.4 (3.5)	0.294
Median CCI (Range)	0 (0–10)	0 (0–6)	0.687
Mean PEG (SD)	4.0 (2.5)	5.8 (2.8)	0.001
**HADS**			
Depression, % (n)	6.3 (7)	22.6 (7)	0.013
Anxiety, % (n)	4.5 (5)	25.8 (8)	0.001
PTSD, % (n)	3.5 (4)	35.5 (11)	< 0.001

HADS, hospital anxiety and depression Scale; PEG, Pain, enjoyment of Life, and general activity Scale; ISS, injury severity Score; PTSD, Post-Traumatic stress syndrome

**Table 3:** Logistic regression results

	OR (95% CI)	*P*-value
Age		
50–64	2.60 (0.55, 12.25)	0.228
65–79	2.52 (0.56, 11.37)	0.230
80+ (reference)	1.00	
Depression	1.11 (0.19, 6.47)	0.909
Anxiety	2.72 (0.47, 15.71)	0.264
PTSD	13.21 (2.90, 60.17)	0.001
PEG	1.33 (1.11, 1.60)	0.002

OR, odds ratio; PTSD, Post-Traumatic stress Syndrome; PEG, Pain, enjoyment of Life, and general activity scale

### Logistic regression results

Results from the logistic regression model are presented in Table 3. Patients with PTSD had significantly higher odds (OR = 13.21, 95% CI = [2.90, 61.17], *p* = 0.001) of having a sleep disturbance compared to those without PTSD. Increasing pain was significantly associated with increasing odds (OR = 1.33, 95% CI = [1.11, 1.60], *p* = 0.002) of having disturbed sleep.

## Discussion

This cross-sectional study examined the patient-reported sleep quality among older adult survivors of trauma and identified an association between symptoms of PTSD, pain, and sleep disturbances in the post-acute period. The prevalence of patient-reported sleep disturbances, defined by a PROMIS-SF score of 14 or more among hospitalized adults and survivors of trauma, was 21.5%. Patients in the 50–64 age group were more likely to report a sleep disturbance, and those with sleep disturbances were more likely to exhibit symptoms of depression, anxiety, PTSD, and pain. These findings highlight the high burden of sleep-related symptoms in this population and emphasize the importance of routine screening and early management of sleep problems following injury.

Our results are consistent with prior studies reporting bi-directional relationships between sleep disturbances and PTSD, anxiety, and depression [5, 21–24]. PTSD can both contribute to and worsen sleep disruption, and inadequate sleep may further exacerbate emotional dysregulation and delayed recovery [35]. These two-way relationships imply that addressing sleep disturbances early after trauma may help improve psychological well-being, while effective treatment of mental health symptoms may enhance sleep quality [36]. Early identification and treatment of PTSD and sleep disturbances thus represent key opportunities to promote holistic recovery in older adults following injury.

Similarly, our findings align with previous evidence linking sleep disturbances and pain, especially in patients recovering from orthopedic trauma [37, 38]. Pain contributes to fragmented and poor-quality sleep, while insufficient sleep can heighten pain sensitivity, creating a self-perpetuating cycle that interferes with rehabilitation and overall recovery [39]. Furthermore, pain has previously been found to have a bidirectional relationship with sleep disturbances in younger populations than the one explored in this study [31, 40, 41]. Therefore, this study extends that evidence to an older trauma population, emphasizing the need for integrated pain and sleep management strategies during hospitalization and early recovery.

Although our results align with previous research, this study provides novel insight into the early post-injury experiences of older trauma survivors. First, it focuses specifically on older adults (≥ 50 years)—a population that is underrepresented in post-injury sleep research and has unique physiological vulnerabilities and recovery trajectories. Second, it captures sleep disturbances during the immediate post-injury hospitalization period, a critical but often overlooked phase when sleep problems are most acute and may influence long-term outcomes. Third, while self-reported sleep assessments have been used in prior studies, our study uniquely integrates PROMIS-based patient-reported outcomes within the Trauma Medical Home (TMH)—a collaborative care model tailored to older trauma survivors. This approach allows for a multidimensional, patient-centered evaluation of the interrelationships between sleep, pain, and psychological symptoms in real-world recovery settings. Together, these elements contribute to a more comprehensive understanding of the biopsychosocial determinants of recovery and identify modifiable risk factors that can be targeted through early, tailored interventions.

Sleep disturbances can significantly affect recovery after an injury by impairing cognitive, psychomotor, mood, cardiovascular, and immunity functions [1, 26, 42, 43]. Early detection of patient-reported sleep disturbances and targeted interventions using validated patient-reported measures such as PROMIS can guide personalized treatment plans, improve resilience, and enhance recovery and quality of life in older injury survivors [43].

### Limitations

This study has several limitations. First, its cross-sectional design can only infer associations, not causalities, between PTSD, pain, and sleep disturbances. Second, the prevalence of sleep disturbance in the study population was lower than that reported in prior studies, which may reflect differences in study populations or measurement timing. Third, data on patients’ preexisting diagnosis of sleep disturbances, pain, or PTSD were not available, precluding adjustment for baseline symptoms. In addition, the study population included participants with an ISS ≥9, denotingmajor trauma, may limit generalizability to patients with milder injuries or to younger populations. Although the bidirectional associations between PTSD and sleep disturbances are well recognized, our analysis examined only one direction– PTSD symptom as predictor of sleep disturbances.

Finally, our study results relied on self-reported measures which may introduce recall bias or reporting error. However, the use of validated, standardized instruments such as PROMIS and HADS strengthens confidence in the accuracy of patient-reported data and underscores the value of incorporating patients’ perspectives to inform early, individualized care.

HADS has been found to be an effective method for surveying depression and anxiety among hospitalized patients, further supporting the reliability of our findings [44].

## Conclusion

Our study identified factors associated with patient-reported sleep disturbances in older injured adults through early screening. These findings highlight the strengths of PROMs in routine screening, guiding tailored interventions to improve sleep quality and associated pain in older injured adults.

## Data Availability

Data is available upon request.

## References

[1] Hillman DR (2021) Sleep loss in the hospitalized patient and its influence on recovery from illness and operation. Anesth Analg 132:1314–1320 33857973 10.1213/ANE.0000000000005323

[2] Jean-Louis G, Kripke DF, Ancoli-Israel S (2000) Sleep and quality of well-being. Sleep 23:1115–1121 11145326

[3] Lu K, Barron JO, Israel H, Cannada LK (2019) Sleep disturbances in orthopaedic trauma patients. OTA Int 2:e040 33937668 10.1097/OI9.0000000000000040 PMC7997085

[4] Wang S, Meeker JW, Perkins AJ et al (2019) Psychiatric symptoms and their association with sleep disturbances in intensive care unit survivors. Int J Gen Med 12:125–130 30962706 10.2147/IJGM.S193084 PMC6434907

[5] Haynes ZA, Collen JF, Poltavskiy EA et al (2021) Risk factors of persistent insomnia among survivors of traumatic injury: a retrospective cohort study. J Clin Sleep Med 17:1831–1840 33928909 10.5664/jcsm.9276 PMC8636356

[6] Thaxton L, Myers MA (2002) Sleep disturbances and their management in patients with brain injury. J Head Trauma Rehabil 17:335–348 12106002 10.1097/00001199-200208000-00007

[7] Wickwire EM, Albrecht JS, Capaldi VF et al (2022) Trajectories of insomnia in adults after traumatic brain injury. JAMA Netw Open 5:e2145310 35080600 10.1001/jamanetworkopen.2021.45310 PMC8792888

[8] Medicine, IoMUCoS (2006) Research a. Sleep Disorders and Sleep Deprivation: An Unmet Public Health Problem 20669438

[9] Watson NF, Badr MS, Belenky G et al (2015) Recommended amount of sleep for a healthy adult: A joint consensus statement of the American academy of sleep medicine and sleep research society. J Clin Sleep Med 11:591–592 25979105 10.5664/jcsm.4758 PMC4442216

[10] Meerlo P, Sgoifo A, Suchecki D (2008) Restricted and disrupted sleep: effects on autonomic function, neuroendocrine stress systems and stress responsivity. Sleep Med Rev 12:197–210 18222099 10.1016/j.smrv.2007.07.007

[11] Finan PH, Quartana PJ, Smith MT (2015) The effects of sleep continuity disruption on positive mood and sleep architecture in healthy adults. Sleep 38:1735–1742 26085289 10.5665/sleep.5154 PMC4813351

[12] Ballard ED, Vande Voort JL, Bernert RA et al (2016) Nocturnal wakefulness is associated with Next-Day suicidal ideation in major depressive disorder and bipolar disorder. J Clin Psychiatry 77:825–831 27337418 10.4088/JCP.15m09943 PMC5103284

[13] Giosan C, Malta LS, Wyka K et al (2015) Sleep disturbance, disability, and post-traumatic stress disorder in utility workers. J Clin Psychol 71:72–84 25099348 10.1002/jclp.22116

[14] Smith MT, Huang MI, Manber R (2005) Cognitive behavior therapy for chronic insomnia occurring within the context of medical and psychiatric disorders. Clin Psychol Rev 25:559–592 15970367 10.1016/j.cpr.2005.04.004

[15] Harvey AG, Jones C, Schmidt DA (2003) Sleep and post-traumatic stress disorder: a review. Clin Psychol Rev 23:377–407 12729678 10.1016/s0272-7358(03)00032-1

[16] Spoormaker VI, Montgomery P (2008) Disturbed sleep in post-traumatic stress disorder: secondary symptom or core feature? Sleep Med Rev 12:169–184 18424196 10.1016/j.smrv.2007.08.008

[17] de Mello MT, Narciso FV, Tufik S et al (2013) Sleep disorders as a cause of motor vehicle collisions. Int J Prev Med 4:246–257 23626880 PMC3634162

[18] Alvaro PK, Roberts RM, Harris JK (2013) A systematic review assessing bidirectionality between sleep Disturbances, Anxiety, and depression. Sleep 36:1059–1068 23814343 10.5665/sleep.2810 PMC3669059

[19] Ramsawh HJ, Stein MB, Belik SL, Jacobi F, Sareen J (2009) Relationship of anxiety disorders, sleep quality, and functional impairment in a community sample. J Psychiatr Res 43:926–933 19269650 10.1016/j.jpsychires.2009.01.009

[20] Germain A (2013) Sleep disturbances as the hallmark of PTSD: where are we now? Am J Psychiatry 170:372–382 23223954 10.1176/appi.ajp.2012.12040432 PMC4197954

[21] Chinoy ED, Carey FR, Kolaja CA, Jacobson IG, Cooper AD, Markwald RR (2022) The bi-directional relationship between post-traumatic stress disorder and obstructive sleep apnea and/or insomnia in a large U.S. Military cohort. Sleep Health 8:606–614 36163136 10.1016/j.sleh.2022.07.005

[22] Germain A, Buysse DJ, Nofzinger E (2008) Sleep-specific mechanisms underlying post-traumatic stress disorder: integrative review and Neurobiological hypotheses. Sleep Med Rev 12:185–195 17997114 10.1016/j.smrv.2007.09.003 PMC2490669

[23] Wafa MH, Viprey M, Magaud L et al (2019) Identification of biopsychosocial factors predictive of post-traUmatic stress disorder in patients admitted to the emergency department after a trauma (ISSUE): protocol for a multicenter prospective study. BMC Psychiatry 19:163 31146712 10.1186/s12888-019-2154-z PMC6543570

[24] Zarzaur BL, Holler E, Ortiz D et al (2024) Collaborative care for injured older adults: the trauma medical home randomized clinical trial. JAMA Surg 159:756–764 38717762 10.1001/jamasurg.2024.1043 PMC11079789

[25] Ortiz D, Meagher AD, Lindroth H et al (2020) A trauma medical home, evaluating collaborative care for the older injured patient: study protocol for a randomized controlled trial. Trials 21:655 32678026 10.1186/s13063-020-04582-x PMC7364470

[26] Gilbert KS, Kark SM, Gehrman P, Bogdanova Y (2015) Sleep disturbances, TBI and PTSD: implications for treatment and recovery. Clin Psychol Rev 40:195–212 26164549 10.1016/j.cpr.2015.05.008 PMC5153364

[27] Charlson ME, Pompei P, Ales KL, MacKenzie CR (1987) A new method of classifying prognostic comorbidity in longitudinal studies: development and validation. J Chronic Dis 40:373–383 3558716 10.1016/0021-9681(87)90171-8

[28] Yu L, Buysse DJ, Germain A et al (2011) Development of short forms from the PROMIS™ sleep disturbance and Sleep-Related impairment item banks. Behav Sleep Med 10:6–24 22250775 10.1080/15402002.2012.636266 PMC3261577

[29] Full KM, Malhotra A, Crist K, Moran K, Kerr J (2019) Assessing psychometric properties of the PROMIS sleep disturbance scale in older adults in independent-living and continuing care retirement communities. Sleep Health 5:18–22 30670160 10.1016/j.sleh.2018.09.003 PMC6347377

[30] Leung YW, Brown C, Cosio AP et al (2016) Feasibility and diagnostic accuracy of the Patient-Reported outcomes measurement information system (PROMIS) item banks for routine surveillance of sleep and fatigue problems in ambulatory cancer care. Cancer 122:2906–2917 27351521 10.1002/cncr.30134

[31] Andersen ML, Araujo P, Frange C, Tufik S (2018) Sleep disturbance and pain: A Tale of two common problems. Chest 154:1249–1259 30059677 10.1016/j.chest.2018.07.019

[32] Zigmond AS, Snaith RP (1983) The hospital anxiety and depression scale. Acta Psychiatr Scand 67:361–370 6880820 10.1111/j.1600-0447.1983.tb09716.x

[33] Lang AJ, Wilkins K, Roy-Byrne PP et al (2012) Abbreviated PTSD checklist (PCL) as a guide to clinical response. Gen Hosp Psychiatry 34:332–338 22460001 10.1016/j.genhosppsych.2012.02.003 PMC3383936

[34] Chen CX, Kroenke K, Stump T et al (2019) Comparative responsiveness of the PROMIS pain interference short forms with legacy pain measures: results from three randomized clinical trials. J Pain 20:664–675 30529442 10.1016/j.jpain.2018.11.010 PMC6551313

[35] Belleville G, Guay S, Marchand A (2009) Impact of sleep disturbances on PTSD symptoms and perceived health. J Nerv Ment Dis 197:126–132 19214048 10.1097/NMD.0b013e3181961d8e

[36] Scott AJ, Webb TL, Martyn-St James M, Rowse G, Weich S (2021) Improving sleep quality leads to better mental health: A meta-analysis of randomised controlled trials. Sleep Med Rev 60:101556 34607184 10.1016/j.smrv.2021.101556 PMC8651630

[37] Swann MC, Batty M, Hu G, Mitchell T, Box H, Starr A (2018) Sleep disturbance in orthopaedic trauma patients. J Orthop Trauma 32:500–504 30086043 10.1097/BOT.0000000000001276

[38] Yang H, Liu YJ, Ye JL, Zhao LH, Li LL, Hou XL (2021) Evaluation of sleep disorder in orthopedic trauma patients: a retrospective analysis of 1129 cases. J Orthop Surg Res 16:344 34051808 10.1186/s13018-021-02487-2 PMC8164244

[39] Mathias JL, Cant ML, Burke ALJ (2018) Sleep disturbances and sleep disorders in adults living with chronic pain: a meta-analysis. Sleep Med 52:198–210 30314881 10.1016/j.sleep.2018.05.023

[40] Freeman R, Wieling W, Axelrod FB et al (2011) Consensus statement on the definition of orthostatic hypotension, neurally mediated syncope and the postural tachycardia syndrome. Clin Auton Res 21:69–72 21431947 10.1007/s10286-011-0119-5

[41] Husak AJ, Bair MJ (2020) Chronic pain and sleep disturbances: A pragmatic review of their Relationships, Comorbidities, and treatments. Pain Med 21:1142–1152 31909797 10.1093/pm/pnz343

[42] Rampes S, Ma K, Divecha YA, Alam A, Ma D (2019) Postoperative sleep disorders and their potential impacts on surgical outcomes. J Biomed Res 34:271–280 32519977 10.7555/JBR.33.20190054 PMC7386412

[43] Feinsilver SH (2021) Normal and abnormal sleep in the elderly. Clin Geriatr Med 37:377–386 34210444 10.1016/j.cger.2021.04.001

[44] Bjelland I, Dahl AA, Haug TT, Neckelmann D (2002) The validity of the hospital anxiety and depression Scale. An updated literature review. J Psychosom Res 52:69–77 11832252 10.1016/s0022-3999(01)00296-3

